# Comparison of Transoral Robotic Surgery and Endoscopic Laryngopharyngeal Surgery for Hypopharyngeal and Supraglottic Laryngeal Cancers

**DOI:** 10.1002/deo2.70178

**Published:** 2025-07-30

**Authors:** Tsutomu Ueda, Takayuki Taruya, Yuji Urabe, Minoru Hattori, Nobuyuki Chikuie, Yuki Sato, Takayoshi Hattori, Hiroaki Tahara, Takao Hamamoto, Takashi Ishino, Sachio Takeno

**Affiliations:** ^1^ Department of Otorhinolaryngology Head and Neck Surgery Graduate School of Biomedical and Health Sciences Hiroshima University Hiroshima Japan; ^2^ Gastrointestinal Endoscopy and Medicine Hiroshima University Hospital Hiroshima Japan; ^3^ Center For Medical Education, Institute of Biomedical & Health Sciences Hiroshima University Hiroshima Japan

**Keywords:** endoscopic laryngopharyngeal surgery | head and neck cancer | hypopharyngeal cancer | supraglottic laryngeal cancers | transoral robotic surgery

## Abstract

**Objectives:**

We aimed to compare intraoperative and postoperative outcomes, technical advantages, and limitations of transoral robotic surgery (TORS) using the Da Vinci Xi robotic system and endoscopic laryngopharyngeal surgery (ELPS) for hypopharyngeal and supraglottic laryngeal cancers.

**Methods:**

This single‐center retrospective cohort study analyzed preoperative variables, intraoperative data, postoperative complications, and functional outcomes in patients with hypopharyngeal and supraglottic laryngeal cancers who underwent TORS or ELPS.

**Results:**

Fifty patients were enrolled (21: TORS; 29: ELPS). Median age at treatment was 73 years (range, 51–87 years). Median resection time was significantly shorter for ELPS (23 min, range 6–124) than for TORS (42 min, range 6–155; *p* < 0.001). No significant association was observed between surgical approach and postoperative complication incidence. Multivariate analysis identified the presence of subepithelial invasion (*p* = 0.0089) as an independent predictor of postoperative complications.

**Conclusion:**

ELPS had a shorter resection time than TORS; however, both approaches showed no significant differences in safety and efficacy.

## 1 Introduction

Transoral robotic surgery (TORS), pioneered by Weinstein et al. and O'Malley et al. [1, 2], has gained significant traction for the treatment of head and neck cancers and has become a popular minimally invasive procedure, particularly for the treatment of patients with early‐stage oropharyngeal cancer. In Japan, the Da Vinci Surgical System was approved by the Pharmaceutical Affairs Agency for oral robot‐assisted surgery to treat laryngopharyngeal cancer in August 2018. The number of TORS cases in Japan has increased, especially since health insurance coverage [3]. TORS for hypopharyngeal and laryngeal cancer is also increasing [4, 5].

In Japan, non‐robotic procedures, endoscopic laryngopharyngeal surgery (ELPS), and transoral video laryngoscopic surgery (TOVS) have been widely utilized for hypopharyngeal and laryngeal cancers, demonstrating their effectiveness [6]. However, there is a lack of comparative studies evaluating its efficacy and safety against non‐robotic procedures such as ELPS for treating hypopharyngeal and laryngeal cancers in Japan. Therefore, this study aimed to evaluate the safety and efficacy of comparing TORS and ELPS techniques.

## 2 Materials And Methods

### 2.1 Study Design and Population

A total of 50 consecutive patients who underwent TORS or ELPS for hypopharyngeal and supraglottic laryngeal cancers between April 2023 and October 2024 were retrospectively enrolled. Eligible patients were aged ≥18 years at the time of resection and had an Eastern Cooperative Oncology Group Performance Status (ECOG PS) of 0–2. Sample size calculation was based on an estimated effect size from previous studies, aiming to achieve sufficient power to detect clinically meaningful differences between TORS and ELPS. Because this investigation was an exploratory retrospective cohort study, no a‐priori power calculation was performed. Instead, all consecutive patients who met the eligibility criteria between April 2023 and October 2024 (*n* = 50) were analysed. This “all‐available‐cases” approach is recommended for exploratory observational research by the Strengthening the Reporting of Observational Studies in Epidemiology guidelines [7].

### 2.2 Examination and Surgical Procedure

All patients were selected based on endoscopic findings and imaging studies, including magnetic resonance imaging, computed tomography, ultrasound, and positron emission tomography scans. The choice of surgical technique (TORS or ELPS) was based on preoperative imaging findings and MDT discussions. TORS is indicated in cases where preoperative biopsy confirms squamous cell carcinoma (SCC) and the mouth is open more than 35 mm. TORS and ELPS were presented, and the procedure was determined by patient choice in cases where TORS was indicated. Neck dissection was either performed simultaneously or prior to resection in cases with positive cervical lymph nodes.

Before lesion resection, an endoscopic examination was conducted under general anesthesia by a team consisting of endoscopists and otolaryngologists for observation and lesion classification. Preoperative depth diagnosis was performed using a combination of oral sonography and narrow‐band imaging as a method that provides high diagnostic accuracy in predicting whether a lesion is a SCC or SCC in situ before treatment, as previously reported [8].

### 2.3 Surgical Technique and Operative Setting

**FIGURE 1:**
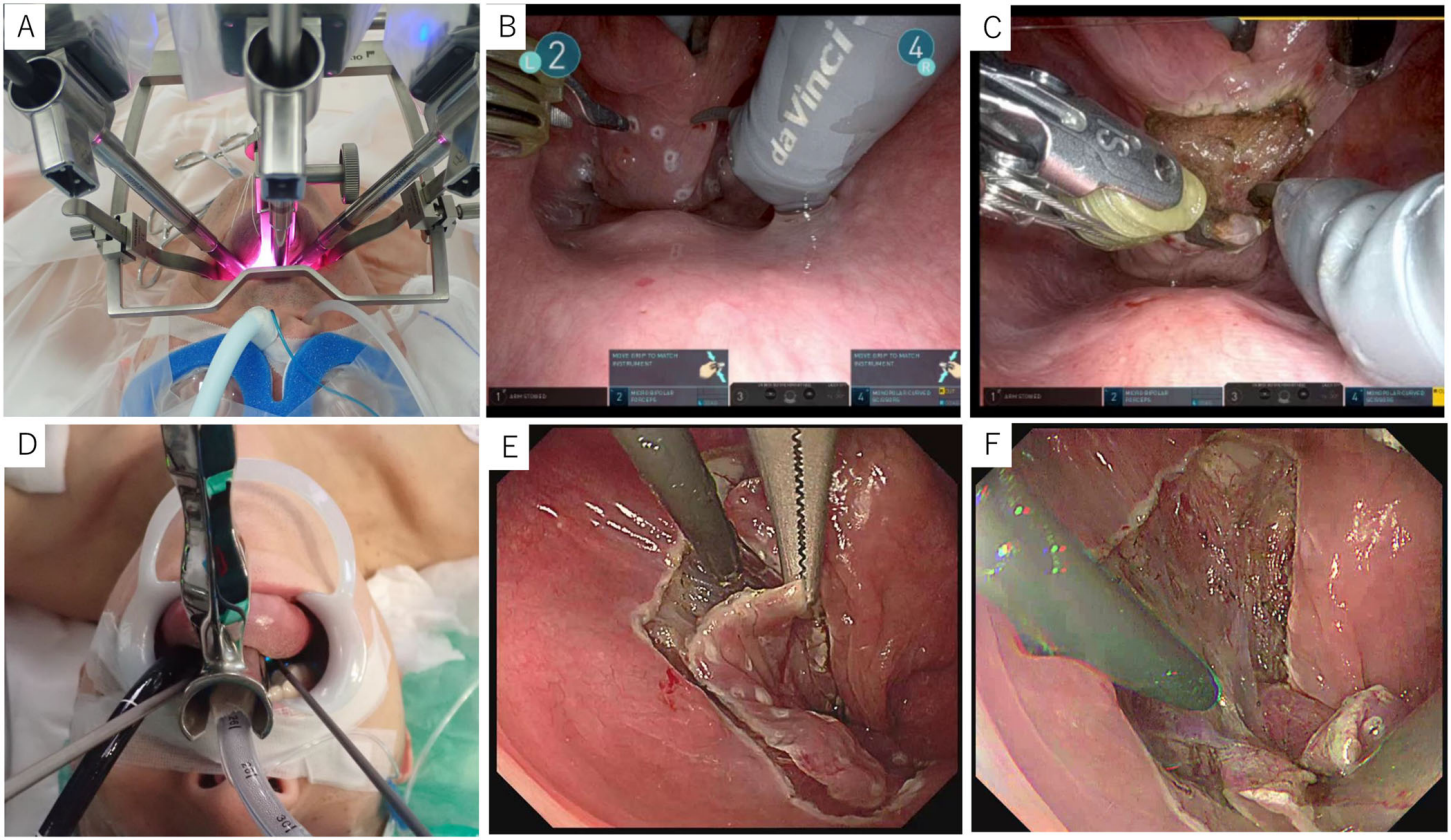
Transoral robotic surgery (TORS) and endoscopic laryngopharyngeal surgery (ELPS) procedures. (A) The 30°endoscopic camera was introduced along the midline of the posterior pharyngeal wall. The remaining two arms were positioned on either side of the camera. The camera was slightly adjusted toward the oral cavity to avoid collision with the other arms, and digital zoom was employed for enhanced visualization. (B) Preoperative marking was performed by endoscopists in cases when lesion identification required more precise endoscopic visualization. (C) The camera was slightly adjusted toward the oral cavity to avoid collision with the other arms, and digital zoom was employed for enhanced visualization. (D) ELPS was performed using Satou's curved laryngoscope. (E) Preoperative marking for all ELPS patients was performed by otolaryngologists. Complete circumferential mucosal resection. (F) The resection in ELPS was performed intraorally with both hands by a head and neck surgeon.

All TORS procedures were performed using the Da Vinci Xi Surgical System (Figure 1A). The supraglottic larynx and hypopharynx were exposed using either the Feyh–Kastenbauer retractor, modified by Weinstein–O'Malley (Olympus, Tokyo, Japan), with the Weinstein–O'Malley TORS blade, or Satou's Curved Laryngo‐Pharyngo Scope with a mouth gag frame (Type‐S2) (Nagashima Medical Instruments).

In some cases, the TROS approach included preoperative marking performed by otolaryngologists, while in others, marking was done by endoscopists prior to the procedure. Preoperative marking was performed by endoscopists in cases where lesion identification required more precise endoscopic visualization (Figure 1B).

The 30° endoscopic camera was introduced along the midline of the posterior pharyngeal wall. The remaining two arms (monopolar curved scissors and micro bipolar forceps) were positioned on either side of the camera. The camera was slightly adjusted toward the oral cavity to avoid collision with the other arms, and digital zoom was employed for enhanced visualization (Figure 1C).

ELPS was performed using Satou's curved laryngoscope (Nagashima Medical Co., Tokyo, Japan) (Figure 1D). This hybrid approach integrates techniques from head and neck surgery and gastrointestinal endoscopy. The resection in ELPS was performed intraorally with both hands by a head and neck surgeon [9]. Unlike TORS, preoperative marking for all ELPS patients was performed by otolaryngologists. (Figure 1E, F)

### 2.4 Endoscopic Examination

All examinations were performed by endoscopists with over 10 years of practical experience. A magnifying endoscope, including the GIF‐H260Z or H290Z (Olympus Medical Systems Co. Ltd), was used for the procedure. Macroscopic types of lesions were categorized using the Japanese Classification of Esophageal Cancer (11th edition) [10], and the microvascular patterns were classified according to the Japanese Esophageal Society classification for Squamous Cell Carcinoma (JES‐SCC classification) [11].

### 2.5 Data Collection and Outcome

The electronic medical records of the included patients were retrospectively reviewed. Patient characteristics included age, sex, smoking history, alcohol consumption, ECOG PS, history of neck irradiation, history of head and neck/esophageal cancers, and use of anticoagulants.

Tumor variables included primary tumor site and subsite, clinical T classification, pathological T classification, macroscopic findings, Presence or absence of subepithelial invasion, tumor thickness, pathological tumor size, resection size, margin status, and p16 status. Other clinical outcomes collected included the total operative duration, console time (TORS only), docking time (TORS only), resection time, operative results, estimated blood loss, postoperative complications, postoperative hospital stay, and time until oral intake after surgery. These variables were selected due to their potential impact on surgical outcomes and postoperative recovery, which are critical to assessing the safety and efficacy of TORS and ELPS. All tumors were staged according to the General Rules for Clinical Studies on Head and Neck Cancer (sixth edition) of the Japan Society for Head and Neck Cancer [12].

The following intraoperative data were also collected: total operative duration, docking time, console time (TORS only), resection time, estimated blood loss, and tracheotomy rate. Docking time was defined as the interval (in minutes) between the initial movement of the robot cart and the final positioning of the last robotic arm into the oral cavity. Console time was measured from the end of docking time until the removal of all robotic arms from the oral cavity.

To compare resection times between TORS and ELPS, we excluded endoscopic observation time from both procedures but included endoscopic marking time in the resection time. Specifically, for ELPS, the resection time was the sum of the marking time and the time from the start of resection to the removal of all instruments from the oral cavity. For TORS, the resection time was the console time, with the endoscopic marking time added when applicable.

Postoperative complications were defined as clinically relevant adverse events (Clavien–Dindo classification) [13] occurring within 30 days of surgery, directly related to the transoral procedure.

### 2.6 Outcomes

The primary endpoint of the study is the comparison of intra‐ and post‐operative outcomes between TORS and ELPS.

Secondary outcomes included a comparison of complications in the two groups, analysis of factors associated with complications, and a learning curve analysis of resection times using the cumulative summation (CUSUM) methodology. To mitigate the potential confounding effect of tumor size on the learning curve, resection times were normalized by tumor volume [14].

### 2.7 Ethical Considerations

This study was approved by the Institutional Review Board of Hiroshima University Hospital (IRB approval number: E‐2039) and was conducted in accordance with the principles of the Declaration of Helsinki. All enrolled patients were informed of the risks and benefits of ELPS and TORS, and written informed consent was obtained from each participant for both study participation and data usage for research purposes.

### 2.8 Statistical Analysis

All statistical analyses were performed using R (version 4.4.2; R Foundation for Statistical Computing, Vienna, Austria). Continuous variables are presented as medians (ranges) and were analyzed using the nonparametric Mann–Whitney U test. Categorical variables are presented as frequencies and percentages and were analyzed using Fisher's exact test. Variables that demonstrated statistically significant associations in univariate analyses were included in a multivariate logistic regression model. Because the objective was exploratory association rather than pre‐operative prediction, the multivariable model incorporated both pre‐ and postoperative covariates (including pathological findings) that could plausibly influence the incidence of complications. Statistical significance was defined as a two‐tailed *p*‐value of <0.05. Because only 20 clinically relevant complication events occurred, the multivariable model was restricted to five a‐priori predictors, yielding an events‐per‐variable ratio of 3.3. Odds ratios are therefore presented as exploratory estimates.

The surgical learning curve was assessed using the CUSUM methodology. The CUSUM method is a sequential analytic technique that tracks performance by plotting the cumulative sum of each observation's deviation from a reference value, such as the overall mean or a predefined target. Recognizing that tumor size is a significant determinant of resection time in TORS, we developed a size‐adjusted performance index. This metric, calculated by dividing tumor size by operative time for each TORS case, represents the rate of tumor resection. This index was then subjected to the same sequential CUSUM analysis.

## 3 Results

Patient characteristics are summarized in Table 1. The predominance of hypopharyngeal lesions (90%) may explain the higher utilization of TORS in this cohort. No significant differences in patient characteristics were observed between the TORS and ELPS groups. No laryngeal hypopharyngeal lesions were resected by endoscopic submucosal dissection (ESD) in the same period.

**TABLE 1:** Patients' characteristics.

	Overall	ELPS	TORS	
Variables	*N* = 50	*N* = 29	*N* = 21	*p*‐Value
Age (y) Median (range)	73 (51–87)	72 (51–86)	75 (51–87)	0.555
Gender (%)				
Female	2 (4)	0 (0)	2 (9.5)	0.171
Male	48 (96)	29 (100)	19 (90.5)	
Primary site (%)				
Hypopharynx	45 (90)	27 (93.1)	18 (85.7)	0.638
Larynx	5 (10)	2 (6.9)	3 (14.3)	
Subsite (%)				
Pyriform sinus	37 (74)	23 (79.3)	14 (66.7)	0.769
Posterior wall	6 (12)	3 (10.3)	3 (14.3)	
Postcricoid area	2 (4)	1 (3.4)	1 (4.8)	
Supraglottis	5 (10)	2 (6.9)	3 (14.3)	
cT (%)				
0	36 (72)	21 (72.4)	15 (71.4)	0.881
1	8 (16)	5 (17.2)	3 (14.3)	
2	5 (10)	2 (6.9)	3 (14.3)	
3	1 (2)	1 (3.4)	0 (0)	
cN (%)				
0	48 (96)	29 (100)	19 (90.5)	0.171
1	2 (4)	0 (0)	2 (9.5)	
p16 (%)				
Negative	47 (94)	27 (93.1)	20 (95.2)	1
Positive	3 (6)	2 (6.9)	1 (4.8)	
ECOG PS (%)				
0	47 (94)	26 (89.7)	21 (100)	0.503
1	2 (4)	2 (6.9)	0 (0)	
2	1 (2)	1 (3.4)	0 (0)	
Smoking (%)				
No	10 (20)	6 (20.7)	4 (19)	1
Yes	40 (80)	23 (79.3)	17 (81)	
Alcohol (%)				
No	1 (2)	0 (0)	1 (5)	0.4
Yes	49 (98)	30 (100)	19 (95)	
History of esophageal cancer (%)				
No	15 (30)	8 (27.6)	7 (33.3)	0.759
Yes	35 (70)	21 (72.4)	14 (66.7)	
History of head and neck cancer (%)				
No	31 (62)	18 (62.1)	13 (61.9)	1
Yes	19 (38)	11 (37.9)	8 (38.1)	
Prior neck radiation therapy (%)				
No	41 (82)	22 (75.9)	19 (90.5)	0.271
Yes	9 (18)	7 (24.1)	2 (9.5)	
Presence of anticoagulants (%)				
No	42 (84)	25 (86.2)	17 (81)	0.706
Yes	8 (16)	4 (13.8)	4 (19)	
Prior treatment (%)				
No	49 (98)	29 (100)	20 (95.2)	0.42
Yes	1 (2)	0 (0)	1 (4.8)	
ME‐NBI (%)				
B1	39 (78)	22 (75.9)	17 (81)	0.377
B2	10 (20)	7 (24.1)	3 (14.3)	
B3	1 (2)	0 (0)	1 (4.8)	
Macroscopic classification (%)				
0‐Ip	1 (2.0)	0 (0)	1 (4.8)	0.15
0‐Is	7 (14.0)	6 (20.7)	1 (4.8)	
0‐IIa	11 (22.0)	4(13.8)	4 (19)	
0‐IIb	28 (56.0)	18 (62.1)	10 (47.6)	
0‐IIc	3 (6.0)	1 (3.4)	2(9.5)	

Transoral surgery was performed on all patients, and surgical margins were negative in all cases. Tracheostomy was performed in one patient in the ELPS group and three patients in the TORS group. All tracheostomy cases involved supraglottic carcinomas, with none in the hypopharyngeal region. No significant difference in the tracheostomy rate was found between the two groups.

The median resection time for the ELPS group (23 min, range 6–124 min) was significantly shorter than that for the TORS group (42 min, range 6–155 min) (*p* < 0.001). No significant difference was observed between the two groups for complication rates. The median time until oral intake after surgery for the ELPS group (3 days, range 1–20 days) was significantly shorter than that for the TORS group (5 days, range 2–20 days) (*p* = 0.038) (Table 2).

**TABLE 2:** Intra‐ and post‐operative outcomes.

	Overall	ELPS	TORS	
Variables	*N* = 50	*N* = 29	*N* = 21	*p*‐Value
Console time (min) Median (range)			71 (24– 140)	NA
Docking time (min) Median (range)			6 (3–13)	NA
Resection time (min) Median (range)	42 (6– 155)	23 (6–124)	80 (29–155)	<0.001
Tumor size (mm) Median (range)	18.5 (4–60)	15 (4.–52)	21 (5–60)	0.178
Resection size (mm) Median (range)	30 (7‐63)	30 (7– 60)	30 (8– 63)	0.768
Blood loss (mL) Median (range)	5 (0–80)	2 (0–50)	7 (0– 80)	0.063
Pathological results (%)				
SCC	15 (30)	9 (31)	6 (28.6)	1
SCC in situ	35 (70)	20 (69)	15 (71.4)	
Tumor thickness (µm)	300 (109–8000)	255 (109–4000)	400 (127–8000)	0.191
Surgical margin (%)				
Negative	50 (100)	29 (100)	21 (100)	NA
Positive	0(0)	0(0)	0(0)	
pT (%)				
0	36 (72)	20 (69)	16 (76.2)	0.533
1	6 (12)	5 (17.2)	1 (4.8)	
2	5 (10)	3 (10.3)	2 (9.5)	
3	3 (6)	1 (3.4)	2 (9.5)	
pN (%)				
0	48 (96)	29 (100)	19 (90.5)	0.171
1	2 (4)	0 (0)	2 (9.5)	
Tracheotomy (%)				
No	46 (92)	28 (96.6)	18 (85.7)	0.297
Yes	4 (8)	1 (3.4)	3 (14.3)	
Period until oral intake after surgery (days) Median (range)	3 (1–20)	3 (1–19)	5 (2–20)	0.038
Postoperative hospital stay (days) Median (range)	8.5 (3–38)	7 (3–22)	11 (5–38)	0.199
Clavien‐Dindo classification (%)				
0	30 (60)	19 (65.5)	11 (52.4)	0.198
I	3 (6)	0 (0)	3 (14.3)	
II	15 (30)	9 (31)	6 (28.6)	
IIIb	2 (4)	1 (3.4)	1 (4.8)	

Table 3 summarizes complications specific to the ELPS and TORS groups. Some patients experienced multiple complications. Overall, 10 of 29 ELPS patients (34.5 %) and 10 of 21 TORS patients (47.6 %) experienced at least one postoperative complication (*p* = 0.39), indicating no statistically significant difference in safety between the two techniques.

**TABLE 3:** Postoperative complications.

Postoperative complications (%)	ELPS (*n* = 29)	TORS (*N* = 21)	*p*‐Value
Any postoperative complication	10 (34.5)	10 (47.6)	0.39
Laryngeal edema	6 (20.7)	6 (28.6)	0.74
Postoperative bleeding	2 (6.9)	1(4.7)	1
Aspiration pneumonia	2 (6.9)	1 (4.7)	1
Swelling of the tongue	0 (0)	3 (14.3)	0.07
Tooth injury	0 (0)	1 (4.7)	0.42
Oral mucosal erosion	0 (0)	1 (4.7)	0.42
Surgical site infection	1 (3.4)	0(0)	1

Univariate analysis revealed that smoking (*p* = 0.037), resection time (*p* = 0.001), tumor size (*p* = 0.045), resection size (*p* = 0.01), estimated blood loss (*p* = 0.007), Tumor thickness (p < 0.001)and presence of subepithelial invasion (*p* < 0.001) were significantly associated with postoperative complications. No significant correlation was found between the surgical procedure and complications (Table 4). Moreover, multivariate analysis identified pathological results (*p* = 0.0089) as an independent predictor for postoperative complications (Table 5).

**TABLE 4:** Univariate analysis of the determinants of postoperative complications.

	Postoperative complications		
	(‐)	(+)	
Variables	*N* = 30	*N* = 20	*p*‐Value
Age (y) Median (range)	74.5 (51–84)	72 (51–87)	0.945
Gender (%)			
Female	0 (0)	2 (10)	0.155
Male	30 (100)	18 (90)	
Primary site (%)			
Hypopharynx	25 (83.3)	20 (100)	0.075
Larynx	5 (16.7)	0 (0)	
Subsite (%)			
Pyriform sinus	20 (66.7)	17 (85)	0.217
Posterior wall	4 (13.3)	2 (10)	
Postcricoid area	1 (3.3)	1 (5)	
Supraglottis	5 (16.7)	0 (0)	
p16 (%)			
Negative	29 (96.7)	18 (90)	0.556
Positive	1 (3.3)	2 (10)	
ECOG PS (%)			
0	30 (100)	17 (85)	0.058
1	0 (0)	2 (10)	
2	0 (0)	1 (5)	
Smoking (%)			
No	9 (30)	1 (5)	0.037
Yes	21 (70)	19 (95)	
Alcohol (%)			
No	0 (0)	1 (5)	0.4
Yes	30 (100)	19 (95)	
History of esophageal cancer (%)			
No	8 (26.7)	7 (35)	0.547
Yes	22 (73.3)	13 (65)	
History of head and neck cancer (%)			
No	20 (66.7)	11 (55)	0.553
Yes	10 (33.3)	9 (45)	
Prior neck radiation therapy (%)			
No	25 (83.3)	16 (80)	1
Yes	5 (16.7)	4 (20)	
Presence of anticoagulants (%)			
No	27 (90)	15 (75)	0.24
Yes	3 (10)	5 (25)	
Prior treatment (%)			
No	30 (100)	19 (95)	0.4
Yes	0 (0)	1 (5)	
Surgical technique			
ELPS	19 (63.3)	10 (50)	0.393
TORS	11 (36.7)	10 (50)	
Resection time (min) Median (range)	30 (6–140)	76.5 (10–155)	0.001
Tumor size (mm) Median (range)	16 (4–60)	23.5 (6–52)	0.045
Resection size (mm) Median (range)	25 (7–63)	38.5 (15–55)	0.01
Estimated blood loss(ml) Median (range)	2 (0–50)	10 (0–80)	0.007
Surgical margin (%)			
Negative	30 (100)	20 (100)	NA
Positive	0 (0)	0 (0)	
Pathological results (%)			
SCC	1 (3.3)	14 (70.0)	<0.001
SCC in situ	29 (96.7)	6 (30.0)	
Tumor thickness (µm)	224.5 (109–1000)	825 (168–8000)	<0.001
Tracheotomy (%)			
No	26 (86.7)	20 (100)	0.14
Yes	4 (13.3)	0 (0)	

**TABLE 5:** Multivariate analysis of the determinants of postoperative complications.

Factor	Reference	Odds ratio (95% CI)	*p*‐Value
Pathological results	SCC in situ	60.0 (4.19–859)	0.0026
Smoking	No	3.00 (0.25–36.10)	0.39
Resection size (mm)	–	1.12 (0.96–1.31)	0.15
Resection time (min)	–	1.04 (0.99–1.09)	0.11
Estimated blood loss (mL)	–	0.99 (0.93–1.05)	0.77
Tumor size (mm)	–	0.85 (0.71–1.02)	0.081

**FIGURE 2:**
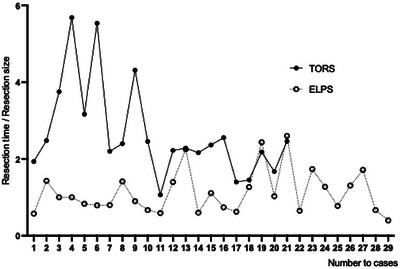
Correlation between number of cases and resection time/resection size ratio. A stable resection time/resection size ratio was achieved for the endoscopic laryngopharyngeal surgery (ELPS) group, while the transoral robotic surgery (TORS) group showed a progressive decrease.

**FIGURE 3:**
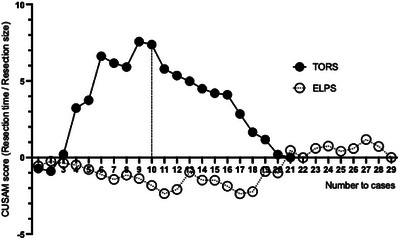
Cumulative summation (CUSUM). In the transoral robotic surgery (TORS) group, the CUSUM score declined after the 10th case.

A learning curve analysis of resection time was conducted for both groups. A stable resection time/resection size ratio was achieved for the ELPS group, while the TORS group showed a progressive decrease (Figure 2). In the TORS group, the CUSUM score declined after the 10th case (Figure 3). In other words, 10 cases were required to achieve stable techniques in TORS for hypopharyngeal laryngeal cancers.

## 4 Discussion

TORS alone has demonstrated favorable postoperative functional outcomes for oropharyngeal cancer [15, 16, 17]. It is also considered advantageous for laryngopharyngeal carcinoma, offering improved functional outcomes and reduced complication rates compared to chemoradiation and radiotherapy [18]. In Japan, non‐robotic surgical procedures, such as ESD, ELPS, and TOVS, are the standard methods for transoral laryngopharyngeal surgery, and their clinical efficacy has been well established [9, 19, 20, 21, 22].

However, these procedures require a high level of manual dexterity and precision, particularly in confined spaces, highlighting the need for the development of novel surgical techniques.

In this study, we compared intraoperative and postoperative outcomes between ELPS and TORS. Significant differences were found in the median resection time and the median period until oral intake, whereas no other significant differences were observed. Regarding complications, only the presence of subepithelial invasion showed a significant correlation, with no notable differences between the surgical procedures. This outcome was linked to the depth of resection, which varied based on the lesion type. For SCC, the resection extended just above the muscle layer. In contrast, for intraepithelial SCC in situ, the resection preserved the subepithelial layer. Importantly, these differences in resection depth were not attributed to the specific surgical technique employed (TOPS or ELPS).

We speculate that the longer median period until oral intake in the TORS group was cautiously resumed due to lack of experience and may be attributed to the frequent tongue and oral cavity complications associated with the retractor, as well as the longer resection time, both of which could potentially delay the resumption of oral intake. However, these complications are expected to decrease due to shorter operating times, and the time to resumption of oral intake is expected to be reduced.

The median resection time for the ELPS group was significantly shorter than that for the TORS group. This finding is consistent with previous studies that have reported shorter resection times in non‐robotic procedures such as ELPS [23]. Given that our institution has performed over 200 ELPS procedures and only around 20 TORS procedures for hypopharyngeal and supraglottic laryngeal cancers, we examined the learning curves for resection time in each surgical approach. While the learning curve for ELPS had already plateaued, the TORS learning curve appeared to level off after approximately 10 cases. Achieving a stable learning curve after approximately 10 cases suggests that surgeons can rapidly gain proficiency with TORS, which may lead to improved surgical outcomes and reduced operative times in subsequent procedures.

Sugishita et al. [24] reported that the number of cases required to acquire a stable technique varied across robot‐assisted surgeries: 14–25 for robot‐assisted gastrectomy, 20–37 for robot‐assisted distal pancreatectomy, 30 for robot‐assisted hepatectomy, and 20 for robot‐assisted pancreaticoduodenectomy. Surgeons with adequate experience in robot‐assisted surgery may acquire stable techniques in a smaller number of cases when transitioning to new robotic procedures.

Our rapid achievement of a stable learning curve, requiring only about 10 TORS cases despite the narrow surgical field, can be attributed to our extensive experience with ELPS and our initiation of TORS for oropharyngeal cancer after completing over 50 procedures by a certain surgeon.

The clear 3D visualization and the absence of endoscope‐induced buffering in TORS make it a highly beneficial technique, substantially reducing the surgeon's burden. In addition, endoscopists are not required at the time of resection, reducing the time burden on the endoscopists. Conversely, we believe that TORS may not be suitable in cases with a limited mouth opening or poor visualization. In such cases, established procedures like ELPS should be considered. In our experience, we attempted TORS in three cases but had to switch to ELPS due to insufficient visualization. ELPS, utilizing a curved laryngoscope that provides a wide field of view and allows the use of curved instruments, is considered a highly advantageous procedure for patients with restricted mouth openings.

This study has some limitations. First, it has its retrospective design and a small sample size from a single institution, which introduces potential selection bias. Although postoperative pathological variables are not available at the time of surgical planning, we included them to identify biological or technical factors that may drive postoperative morbidity. The present findings should therefore be interpreted as descriptive; future work aimed at pre‐operative risk stratification should rely solely on variables available before surgery. Second, the multivariable analysis is limited by a low events‐per‐variable ratio (<10), which increases the risk of type II error and results in wide confidence intervals. Consequently, results such as the elevated odds ratio for smoking should be viewed as preliminary and validated in larger studies. Prospective, multicenter studies that account for patient selection are warranted in the future. Further studies are needed to explore the long‐term functional outcomes of TORS and ELPS in this patient population, including quality of life measures such as swallowing function, speech, and the need for adjuvant treatments like radiotherapy.

This study demonstrated that ELPS and TORS are both safe and effective surgical options for hypopharyngeal and laryngeal cancers, with ELPS showing shorter resection times but no significant differences in overall safety or efficacy. Given the learning curve associated with TORS, a reduction in resection time is expected as surgical proficiency improves, which may enhance the overall efficiency and effectiveness of the procedure over time. Additionally, TORS, with its delicate manipulation and clear 3D visualization, is anticipated to gain popularity in the future, while the indication for ELPS could be limited to patients with restricted mouth opening. Further studies with larger sample sizes and long‐term functional outcomes are needed to better evaluate the advantages and limitations of TORS and ELPS in treating hypopharyngeal and laryngeal cancers.

## Ethics Statement

**Approval of the research protocol by an Institutional Review Board**: This study was approved by the Institutional Review Board of Hiroshima University Hospital (IRB approval number: E‐2039) and was conducted in accordance with the principles of the Declaration of Helsinki.

## Consent

All enrolled patients were informed of the risks and benefits of ELPS and TORS, and written informed consent was obtained from each participant for both study participation and data usage for research purposes.

## Conflicts of Interest

The authors declare no conflicts of interest.

## Data Availability

The data that support the findings of this study are available from the corresponding author upon reasonable request. N/A
